# Protective effect of crocin on hemodynamic parameters, electrocardiogram parameters, and oxidative stress in isolated hearts of rats exposed to PM_10_

**DOI:** 10.22038/IJBMS.2022.61163.13533

**Published:** 2022-04

**Authors:** Esmat Radmanesh, Mahin Dianat, Mohammad Badavi, Gholamreza Goudarzi, Seyyed Ali Mard, Maryam Radan

**Affiliations:** 1 Research Center for Environmental Contaminants (RCEC), Abadan University of Medical Sciences, Abadan, Iran; 2 Persian Gulf Physiology Center, Medical Basic Sciences Research Institute, Ahvaz Jundishapur University of Medical Sciences, Ahvaz, Iran; 3 Department of Physiology, Faculty of Medicine, Ahvaz Jundishapur University of Medical Sciences, Ahvaz, Iran; 4 Air Pollution and Respiratory Diseases Research Center, Ahvaz Jundishapur University of Medical Sciences, Ahvaz, Iran

**Keywords:** Crocin, Electrophysiological factors, Hemodynamic parameters, Oxidative stress, Particulate matter

## Abstract

**Objective(s):**

Exposures to particulate matter (PM) have been related to increased risk for cardiovascular health effects and can promote cardiac ischemia and oxidative stress. Crocin has strong antioxidant properties and stress-reducing effects. Therefore, this study considered the effect of crocin on cardiovascular parameters in rats exposed to PM_10_.

**Materials and Methods:**

Forty Wistar rats (male, 250–300 g) were grouped as control, receiving normal saline and crocin, receiving PM_10_, receiving PM_10_+Crocin. Instillation of PM_10_ into the trachea was done. Forty-eight hours after exposure to the normal saline or PM, the heart was separated. Hemodynamic and electrophysiological factors were measured. The levels of superoxide dismutase (SOD), glutathione peroxidase (GPx), catalase activity (CAT), malondialdehyde (MDA), xanthine oxidase, were evaluated by kits.

**Results:**

The voltage of the QRS complex was significantly reduced and PR and QTc intervals increased in PM_10_ groups. Hemodynamic parameters before ischemia and in the ischemic-reperfusion stage, in the PM_10_ group, showed a significant decrease. In the ischemic hearts of the PM_10_ group, a significant decline in the activity of CAT, SOD, and GPx, and a significant increase in MDA and XOX enzymes activity were observed, and crocin improved all of these factors.

**Conclusion:**

Cardiac ischemia causes abnormal hemodynamic factors of the heart, which are exacerbated by PM_10_ and further reduce the heart’s contractile strength. Increased oxidative stress due to PM_10_ is probably one of the important reasons for these changes. This study suggests that the use of antioxidants such as crocin improves the cardiovascular adverse effects of myocardial ischemia and PM_10_ exposure.

## Introduction

Particulate matter (PM) characterizes a compound mixture of inorganic and organic substances and a mixture of liquid and solid particles suspended in the air that vary in origin, size, and composition (1). Exposures to the particulate matter have been linked with augmented risk for cardiovascular health effects and are related to acute ischemic, myocardial infarction, stroke, atherosclerosis, and death (2-4). The prevalence of cardiac arrest, myocardial infarction, ischemic heart disease, and heart failure increases with increasing dust exposure (5-8). For the pathophysiological actions and toxicity of PM on the cardiovascular system, oxidative stress, an imbalance between the anti-oxidants defenses and the generation of reactive oxygen species (ROS) is the primary mechanism. Cell death and inflammation can result from oxidative damage to proteins, lipids, and DNA if anti-oxidant defenses are not sufficient to prevent cell damage. (9-10). In polluted urban areas, anti-oxidant-rich foods may be protective of cardiovascular health (11).

Crocin acts is an anti-oxidant that is a unique water-soluble carotenoid and active monomer extracted from *Crocus sativus* L. (saffron). It has been reported to have some beneficial properties, such as cardiovascular protective effects, for the treatment of myocardial ischemia and hypoxia. It improves behavior and cognition; It is and anti-lipid, anti-atherosclerotic and prevent the radicals from slipping and inhibits the activity of free radicals (12-19).

Considering the destructive effects of PM on human health, especially cardiovascular health, and since the cultivation of medicinal plants in Iran is widespread and on the other hand, the complications and toxicity of medicinal plants compared with synthetic chemical drugs is negligible and in many cases can be neglected; also, as it has been stated in previous studies, crocin is one of the most effective components of saffron with anti-oxidant and stress-reducing effects. Therefore, this study investigated the effect of crocin on cardiovascular parameters in rats exposed to PM.

## Materials and Methods

***Place of PM***_10_ ***sampling***

Sampling for this study was done in Ahvaz, located in a dry region with high humidity and temperature, low vegetation, and high winds in southwestern Iran. There are many deserts around the region, especially in the west and neighboring countries such as Iraq and Saudi Arabia, which have been identified as the primary dust sources for the city (20-21).

Sampling was performed on the roof of Ahwaz School of Health and at a distance of 10 meters from the ground for 24 hr. In this study, according to the objectives, the measurement of suspended particles smaller than 10 microns (PM-10) was considered. The device used in this study was a US-made High volume PM_10_ sampler (Tisch company) used by the United States Environmental Protection Agency (USAID) to sample airborne particles.The sampler had a quartz filter (22-23). 

***Animals and grouping***

Forty Wistar rats (male, weighing 250 to 300 g) were randomly selected. Animals were kept in standard cages in a room with relative humidity, a temperature of 22 °C, ventilation, and a twelve-hour/twelve-hour light/dark cycle with permitted access to adequate water and food. Animals underwent deep anesthesia at all stages of surgery. There were also ethical considerations such as heating and cooling conditions, air, light and breathing space, and adequate space and cages for animal species. Ethical Approval reference number was IR.AJUMS.REC.1395.419 . Grouping is as follows: control group, group receiving normal saline (0.1ml, intratracheal installation) (22) and crocin (50 mg/kg, intraperitoneal) (24), group receiving PM_10_ (5 mg/kg mixed with 0.1ml normal saline, intratracheal installation) (22), group receiving PM_10_ (5 mg/kg, mixed with 0.1 ml normal saline, intratracheal instillation) + Crocin (50 mg / kg, intraperitoneal).

***Exposure to air pollution***

Removal of PM_10_ from the filter surface was performed with a scalpel (25) and prepared as a suspension in saline (0.1 ml), and was mixed for twenty min (26, 27). Animal anesthesia was achieved with 50 mg/kg ketamine and 5 mg/kg xylazine. Instillation of PM_10_ into the trachea was done in two stages. Then the rats were connected to a ventilator for five min. Forty-eight hours after exposure to the animal for normal saline or PM, rats were again anesthetized. They were exposed to the suspension for the second time. After half an hour, crocin was administered intraperitoneally, and after half an hour the heart was separated and the hemodynamic parameters were measured.

***Blood pressure recording method***

Systolic blood pressure (SBP) was recorded before and after receiving PM, crocin, and normal saline by tail-cuff 30 min after rats were anesthetized using Power Lab apparatus (AD-Instruments, Australia).

***Electrocardiographic recording method***

Before and after receiving PM_10_ and crocin in all groups, 30 min after anesthesia by 10 mg/kg xylazine and 50 mg/kg ketamine through the intraperitoneal (IP) route, to evaluate the effective voltage of QRS, determine the QT interval and P-R interval was recorded using lead II and Bio Amp. ECG was monitored by a Power Lab apparatus. Bazzet’s formula is a formula to correct QT for heart rate (QTc):

Bazzet’s formula: QTc (QT corrected for HR) = QT/square root RR

***Heart separation method***

Rats were anesthetized using ketamine/xylazine and heparin. To prevent coagulation, 1,000 units heparin per kilogram of animal weight was injected intraperitoneally. After the animal is attached to the ventilator, breathing will be done in the air; after opening the abdominal cavity, the ruptured aperture and chest are returned. By inserting a shear into the aorta, a metal cannula wase inserted into it and fixed with a yarn (aortic cannulation). Then the hearts of the animals were separated and transferred to the Langendorff machine containing the Krebs-Hansel solution. The tissue dissolved by this solution is continuously fed by the peristaltic pump. After transferring the heart to the Setup Langendorff, the heart is allowed for a period of 15 min to adapt to the new conditions, and after the heart is stable, it is transferred to the power lab to measure the parameters, and is monitored and recorded during the test (28). Rats are anesthetized with ketamine/xylazine. After connecting to a ventilator, and opening the abdominal cavity, the ruptured diaphragm and chest return. By placing an incision on the aorta, aortic cannulation was performed. The hearts of the rats were separated and transferred to a Langendorf apparatus containing Krebs-Hansel solution (28).

***Measurement of hemodynamic parameters of the heart***

A balloon filled with water enters the pressure transducer into the left ventricle via the left atrium. The heart is housed in a double-walled glass chamber in which the temperature can be controlled for 30 min to standardize heart function. The volume of the balloon is changed so that the final diastolic pressure is equal to 5 mm Hg and the signal received by the pressure transducer is analyzed by the power lab system, and the following parameters are measured (29): Left ventricular systolic pressure (LVSP), Left ventricular end-diastolic pressure (LVEDP), Left ventricular developed pressure (LVDP = LVSP - LVEDP), Maximum rate of rising (+ dp/dt), Maximum rate of fall (-dp/dt) as the indexes of contraction and relaxation. Throughout the test, heart rate and perfusion pressure will be controlled (22).

***Ischemic-reperfusion protocol***

In the isolated heart, it is initially considered for 30 min to stabilize left ventricular pressure and coronary perfusion pressure (CPP), and for complete perfusion anesthesia, it will be cut off for 30 min, and the perfusion will be restored for 60 min (22).

***Biochemical tests***

After performing the previous steps mentioned, the heart tissue was kept at a temperature of 80 °C for biochemical measurements. To evaluate the level of catalase activity (CAT), superoxide dismutase (SOD), malondialdehyde (MDA), glutathione peroxidase (GPx), and xanthine oxidase are used by a kit from Zell Bio GmbH Company (Germany). 

***Statistical methods analysis of results***

Data analysis was conducted with GraphPad Prism 6 (GraphPad Software, La Jolla, CA). All data are presented as mean ± SEM. Descriptive statistics were performed to obtain baseline characteristics. Quantitative variables distribution was tested with Kolmogorov-Smirnov normality test. Parametric (One-way ANOVA and repeated measures ANOVA) and nonparametric tests (Kruskal-Wallis and Friedman) were applied to describe the differences between groups for the variables of interest as appropriate. Results followed by LSD were used for multiple comparison tests. *P*<0.05 was considered statistically significant.

## Results

***Blood pressure***

**Figure 1:**
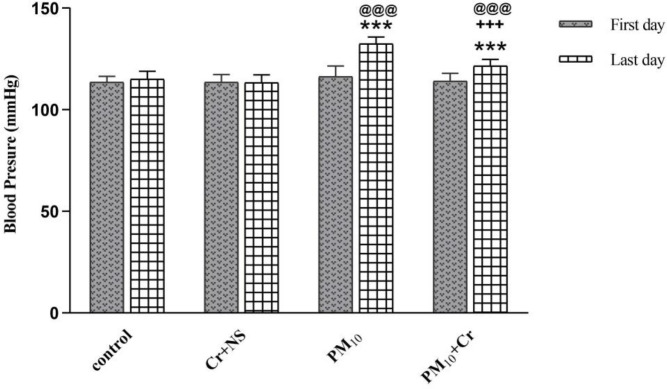
Effect of PM_10_ and crocin on blood pressure on the first day and last day. Control group, Cr +NS, PM_10_ group, PM10 + Cr group. ***P<0.001 vs control group, +++ *P*<0.001 vs PM_10_ group, @@@ *P*<0.001 before vs after. Repeated measurement ANOVA was used followed by the LSD test

Blood pressure in the groups receiving PM_10_ and PM_10_ + crocin 48 hr after receiving PM_10_ revealed a significant increase compared with the first day and also showed a significant increase compared with the control group (*P*<0.001). The PM_10_ + crocin group revealed a significant decrease compared with the PM_10_ group (*P*<0.001) (Figure 1).

***Cardiac electrophysiological parameters***

**Figure 2:**
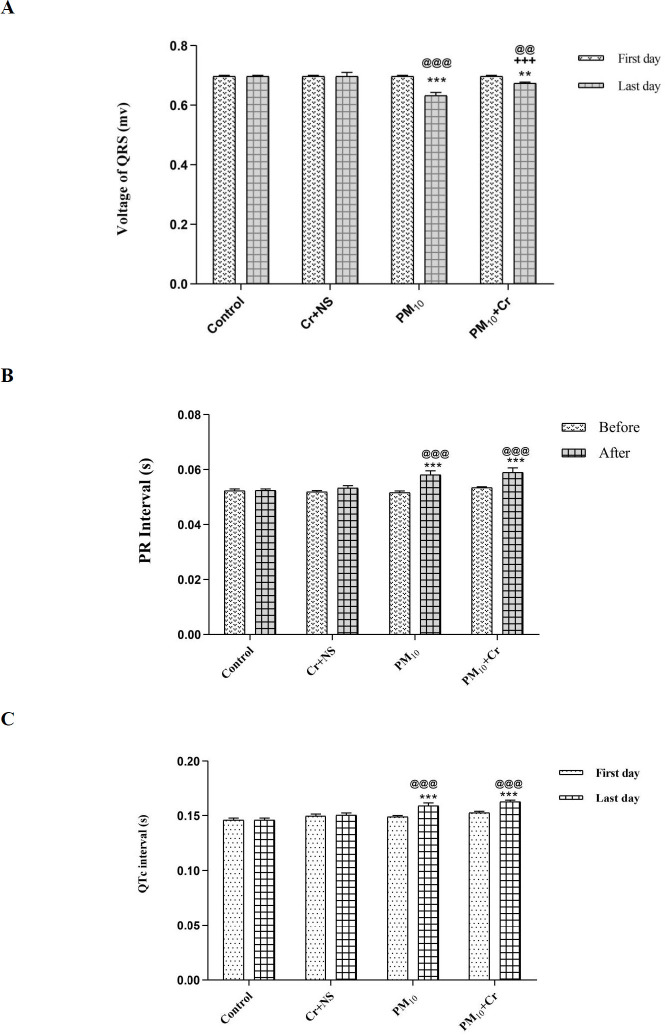
Effect of PM_10_ and crocin on electrocardiogram parameters A: voltage of QRS, B: PR interval, and C: QTc interval on first and last days. Control group, Cr +NS, PM_10_ group, PM10 + Cr group. Repeated measurement ANOVA was used followed by the LSD test. **P<0.01, ***P<0.001 vs Control group. +++ *P*<0.001 vs PM10 group. @@ *P*<0.01, @@@ *P*<0.001 before vs after

QRS: QRS Complex; QTc: QT interval corrected; Cr: Crocin; NS: Normal Saline; LSD: Least Significant Difference

QRS voltage at 48 hr after receiving PM_10_ in PM_10_ and PM_10_ + crocin groups decreased significantly compared with the first day (*P*<0.001 and *P*<0.01, respectively). QRS voltage compared with the control group, in the PM_10_ and PM_10_ + crocin groups decreased significantly (*P*<0.001 and *P*<0.01, respectively), and in the PM_10_ + crocin group compared with the PM_10_ group increased significantly (*P*<0.001) (Figure 2A).

PR-interval in the PM_10 _and PM_10_ + crocin groups, 48 hr after receiving PM_10_ showed a significant increase compared with the first day (*P*<0.001), and a significant increase was seen in the PM_10 _ and PM_10_ + crocin groups compared with the control group (*P*<0.001) (Figure 2B).

QTc-interval forty-eight hours after receiving PM_10_ had a significant increase in the PM_10 _and PM_10_ + crocin groups compared with the first day (*P*<0.001). Also, QTc-interval in the PM_10_ and PM_10_ + crocin groups compared with the control group increased significantly (*P*<0.001) (Figure 2C).

***Hemodynamic parameters***

*LVSP*

LVSP in the pre-ischemic time in the PM_10_ group compared with the control group decreased significantly (*P*=0.007). In the first 20 min of reperfusion, a significant decrease in LVSP was seen in all groups compared with before ischemia. At this time, LVSP decreased significantly in the group receiving PM_10_ compared with the control group (*P*<0.001) and increased significantly in the PM_10_ + crocin group compared with the PM_10_ group (*P*<0.01).

**Figure 3:**
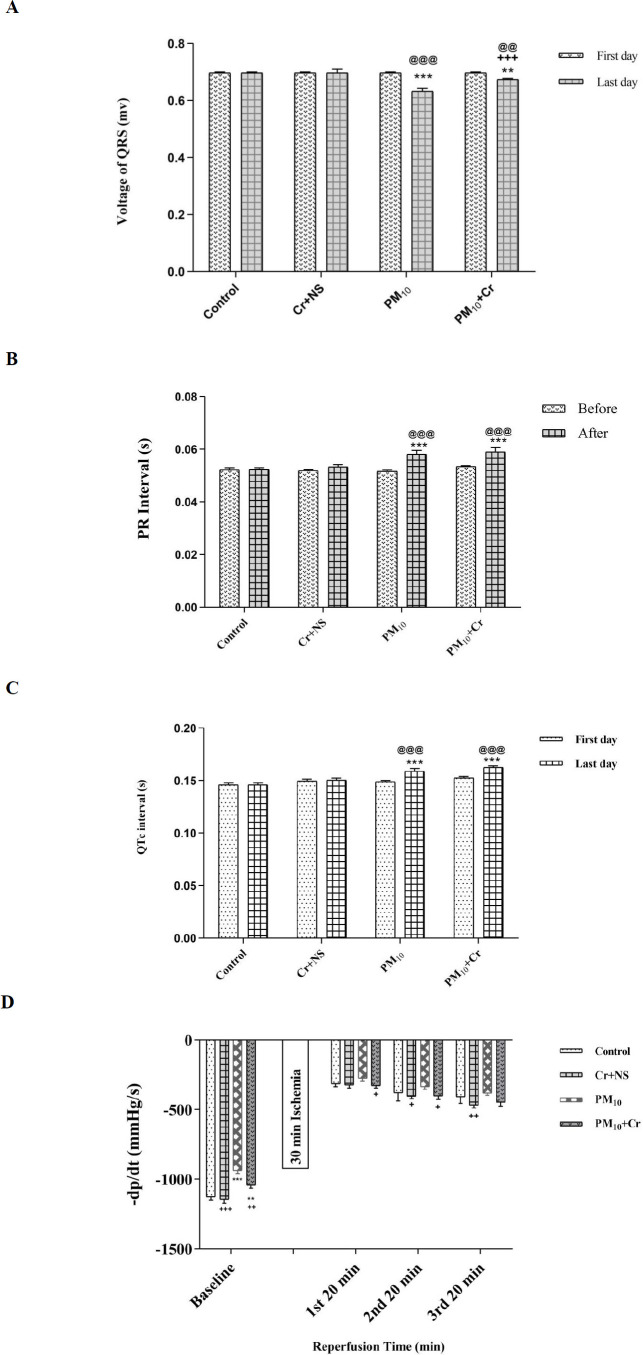
Effect of PM_10_ and crocin on A: LVSP, B: LVDP, and C&D: ±dp/dt. Control group, Cr +NS, PM_10 _group, PM_10_ + Cr group. Repeated measurement ANOVA was used followed by the LSD test. *P<0.05, **P<0.01, and ***P<0.001 vs Control group. +*P*<0.05, ++*P*<0.01, +++ *P*<0.001 vs PM_10_ group

In the second 20 min of reperfusion, LVSP decreased significantly in the PM_10_, and PM_10_ + crocin groups compared with the control group (*P*<0.001 and *P*<0.01, respectively), and increased significantly in PM_10_ + crocin receiving group compared with the PM_10_ receiving group (*P*<0.01) (Figure 3A).

*LVDP*

LVDP decreased significantly in the PM_10_ group compared with the control group before ischemia (*P*<0.01). LVDP increased in the PM_10_ + crocin group compared with the PM_10_ group significantly (*P*<0.001). After ischemia, LVDP decreased significantly in all groups compared with before ischemia. In the first 20 min of reperfusion, LVDP decreased significantly in the PM_10_ group compared with the control group (*P*<0.001). There was also a significant increase in the crocin receiving group compared with the control group (*P*<0.001). LVDP increased in PM_10_ + crocin group compared with the PM_10_ group significantly (*P*<0.01), and in the second 20 min of reperfusion, LVDP decreased significantly in the PM_10_ group compared with the control group (*P*<0.001). A significant increase was seen in LVDP in the crocin group compared with the control group (*P*<0.001) and increased in PM_10_ + crocin group compared with the PM_10_ group significantly (*P*<0.01). In the third 20 min of reperfusion, LVDP decreased significantly in the PM_10_ group compared with the control group (*P*<0.01) and increased significantly in the crocin group compared with the control group (*P*<0.001). There was a significant increase in PM_10_ + crocin receiving group compared with the PM_10_ group (*P*<0.001) (Figure 3B).

*+ dp/dt*

Before ischemia, + dp/dt decreased significantly in the PM_10_ group compared with the control group (*P*<0.01) and increased significantly in the PM_10_ + crocin group compared with the PM_10_ group (*P*<0.01). In all groups, + dp/dt decreased significantly after ischemia compared with before ischemia. In the first twenty min of reperfusion, a significant decrease was seen in the PM_10_ group compared with the control group (*P*<0.01). + dp/dt increased significantly in the PM_10_ + crocin group compared with the PM_10_ group (*P*<0.001). In the second twenty min of reperfusion, a significant decrease was seen in the PM_10_ group compared with the control group (*P*<0.01). + dp/dt increased significantly in the PM_10_ + crocin group compared with the PM_10_ group (*P*<0.001). In the third twenty min of reperfusion, + dp/dt decreased significantly in the PM_10_ group compared with the control group (*P*<0.01), and A significant increase was seen in the PM_10_ + crocin group compared with the PM_10_ group (*P*<0.001) (Figure 3C).

*-dp/dt*

In the time before ischemia, -dp/dt decreased significantly in the group receiving PM_10_ (*P*<0.001) and PM_10_ + crocin (*P*<0.01) compared with the control group and increased in the PM_10_ + crocin group compared with the PM_10_ group significantly (*P*<0.01). In all groups, a significant decrease in - dp/dt was seen after ischemia compared with before ischemia. In the first twenty min of reperfusion, -dp/dt increased significantly in the PM_10_ + crocin group compared with the PM_10_ group (*P*<0.05). In the second twenty min of reperfusion, a significant increase was seen in the PM_10_ + crocin group compared with the PM_10_ group (p<0. 05) (Figure 3D).

*CAT activity*

**Figure 4:**
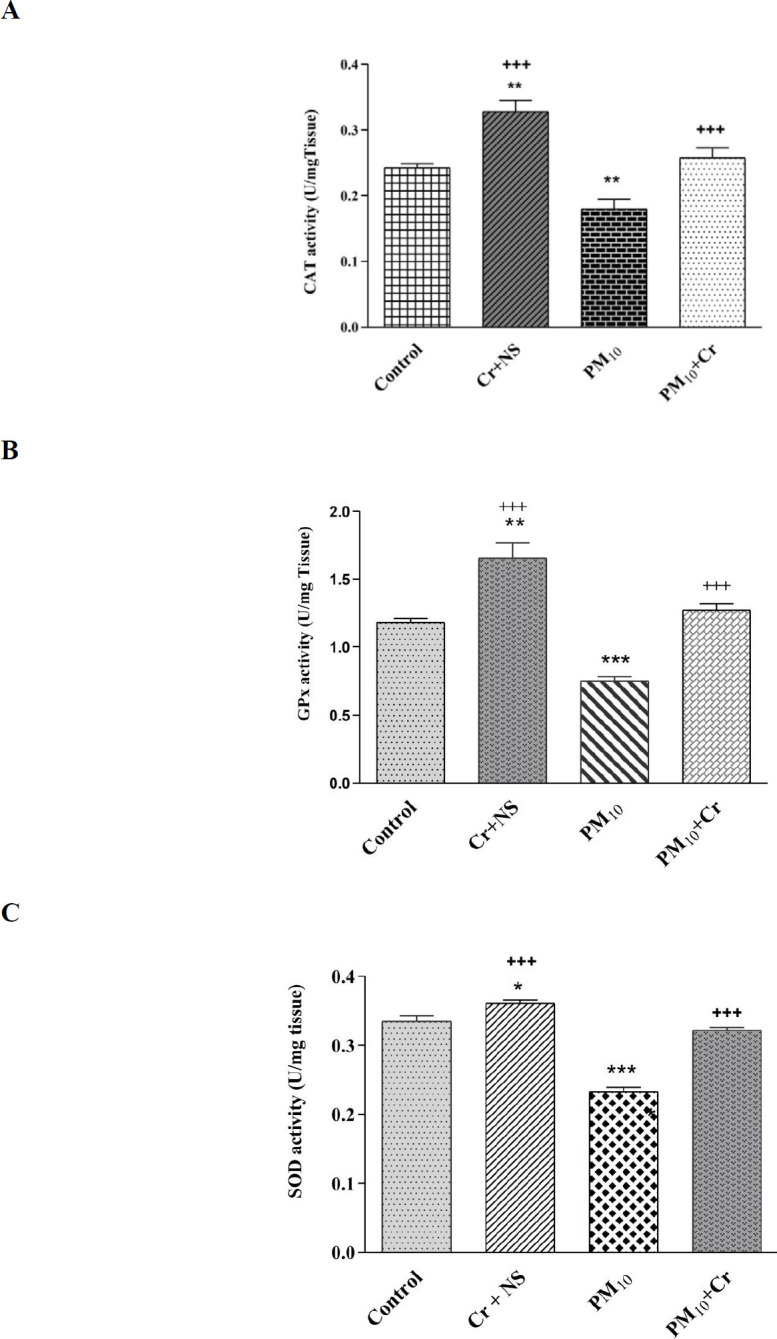
Effect of PM_10 _and crocin on A: CAT, B: GPx, and C: SOD. Results are expressed as mean ± SEM. Control group, Cr +NS, PM_10_ group, PM_10 _+ Cr group. One-way ANOVA was used followed by the LSD test. *P<0.05, **P<0.01, and ***P<0.001 vs Control group. +++ *P*<0.001 vs PM_10 _group

CAT activity decreased significantly in the PM_10_ group compared with the control group (*P*<0.01) and increased significantly in the crocin group compared with the control group (*P*<0.01). CAT activity increased in the PM_10_ + crocin group compared with the PM_10_ group significantly (*P*<0.001) (Figure 4A).

*GPx activity*

GPx activity decreased in the PM_10_ group compared with the control group significantly (*P*<0.01) and increased significantly in the crocin group compared with the control group (*P*<0.001). GPx activity increased in the PM_10_ + crocin group compared with the PM_10_ group significantly (*P*<0.001) (Figure 4B).

*SOD activity*

A significant decrease in SOD activity was seen in the PM_10_ group compared with the control group (*P*<0.001) and a significant increase in the crocin group compared with the control group (*P*<0.05). SOD activity increased in the PM_10_ + crocin group compared with the PM_10_ group significantly (*P*<0.001) (Figure 4C).

*XOX activity*

**Figure 5:**
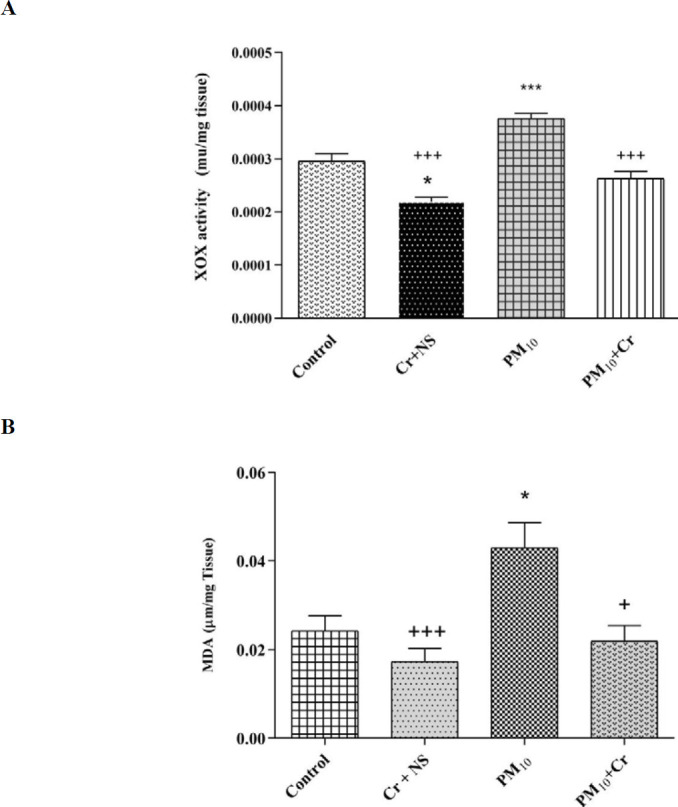
Effect of PM_10_ and crocin on A: MDA and B: XOX. Results are expressed as mean ± SEM. Control group, Cr +NS, PM_10 _group, PM_10_ + Cr group. One-way ANOVA was used followed by the LSD test. *P<0.05, ***P<0.001 vs Control group. +*P*<0.05, +++ *P*<0.001 vs PM_10_ group

XOX activity increased in the PM_10_ group compared with the control group significantly (*P*<0.001) and decreased in the crocin group compared with the control group significantly (*P*<0.05). XOX activity decreased in the PM_10_ + crocin group compared with the PM_10_ group significantly (*P*<0.001) (Figure 5A).

*MDA*

MDA increased in the PM_10_ group compared with the control group significantly (*P*<0.05). There was a decrease in MDA in the crocin group compared with the control group, but this decrease was insignificant. MDA decreased in the PM_10_ + crocin group compared with the PM_10_ group significantly (*P*<0.05) (Figure 5B).

PM10: particles with aerodynamic diameter<10 μm

Cr: Crocin; NS: Normal Saline; LSD: Least Significant Difference

PM_10_: particles with aerodynamic diameter<10 μm; LVSP: left ventricular systolic pressure; LVDP: left ventricular developed pressure

Cr: Crocin; NS: Normal Saline; LSD: Least Significant Difference

PM_10_: particles with aerodynamic diameter<10 μm; CAT: catalase

Cr: Crocin; NS: Normal Saline; LSD: Least Significant Difference; SOD: superoxid dismutase; GPx: glutathion peroxidase

PM_10_: particles with aerodynamic diameter<10 μm; MDA: malondialdehyde

Cr: Crocin; NS: Normal Saline; LSD: Least Significant Difference; XOX: xanthine oxidase

## Discussion

In this study, the voltage of QRS was significantly reduced in the groups receiving PM_10_. PR interval and QTc interval in PM_10_ groups showed a significant increase. Hemodynamic factors such as LVSP, LVDP, and ± dp/dt, before ischemia in the PM_10_ group, showed a significant decrease. After ischemia, a significant decrease was detected in all groups compared with before ischemia. In the ischemic-reperfusion stage, a significant decrease in LVDP, LVSP, dp/dt_max_, and dp/dt_min_ was observed in the PM_10_ group. These results indicate that the role of PM in reducing the contractile strength of the heart and crocin in the PM_10_ + crocin receiving group improved all of these factors.

In the study of Peng *et al*., a significant positive association was found between hospitalization due to peripheral vascular diseases and daily changes in air pollution (29). In the Peters study, there was an association between the daily changes in free air suspended particulate concentrations and cardiovascular mortality, hospital admissions, and exacerbations of cardiovascular disease symptoms (30). Exposure to PM_2.5_ is associated with augmented collagen deposition and loss of cardiac contractility in healthy rats (31). Exposure to PM_2.5_ was associated with an augmented S-T segment depression and increased levels of malondialdehyde (32). The risk of myocardial infarction is related to the oxidative potential of PM_2.5 _(33). Exposure to PM_2.5 _impaired left ventricular function, and AMP-activated protein kinase was involved in oxidative stress (34).

In our study, systolic blood pressure increased in the groups receiving PM_10_ significantly. Other studies reported that ambient air pollution was associated with greater hypertension prevalence and raised blood pressure (35-36). An increase in blood pressure of 10 mm Hg was observed after nine months of PM2.5 exposure, which was associated with decreased contraction and ventricular regeneration (37). Exposure to PM_2.5 _increases vasoconstrictive pathways while reducing vasodilating capacity and disrupting vascular function (38).

In this study, crocin improved systolic blood pressure in the group receiving PM10 + crocin. Shahidi reported that the aqueous extract of saffron in healthy and high blood pressure rats has an effect of reducing blood pressure (39).

In the ischemic tissue of the heart of the PM_10_ group, a significant decline in the activity of CAT, SOD, and GPx, as anti-oxidant enzymes, was perceived. MDA and XOX enzyme activity in the group receiving PM_10_ significantly increased, which indicates the role of PM in oxidative stress. In several human studies, the relationship between dust and oxidative stress has been observed, and with increasing air pollution, the increase in biological markers of protein, lipid, and DNA oxidation was shown (40-42). Exposure to ozone for 1–2 months modified left ventricular function, which was related to declines in SOD activity, increases in lipid peroxidation, and cytokines in the heart (43). Nine months of PM2.5 exposure reduced the total capacity of plasma anti-oxidants (37). The stimulation of oxidative stress by PM is associated with their physical and chemical properties. PM can interfere with the signaling pathways that lead to ROS production, with ROS-producing organs such as mitochondria (44).

In this study, the activity of anti-oxidants enzymes (GPx, SOD, and CAT) decreases significantly in the ischemic heart tissue of PM_10_ group and crocin group, which proves crocin’s anti-oxidant role. Crocin in the PM_10_ + crocin group improved the activity of these anti-oxidant enzymes. The activity of MDA and XOX enzymes in the crocin receiving group, significantly decreased, which indicates the role of the crucial crocin anti-oxidant. Crocin in the PM_10_ + crocin group reduced the activity of these two enzymes. Oxidative stress was strangely suppressed by crocin administration reflected by repressed MDA along with enriched reductive/anti-oxidative power in ischemia-reperfusion rats (45-46). In diabetic rats treated with crocin it was observed that GSH levels increased significantly and MDA levels decreased in heart tissue and serum and total cholesterol, LDL, and TG concentrations decreased, and HDL levels in serum increased (46).

In this study, crocin in the PM10 + crocin group improved hemodynamic factors such as LVSP, LVDP, and ± dp/dt before ischemia and in the ischemic-reperfusion stage. In a study conducted by Guantan and colleagues in 2014 on rats, it was observed that crocin (20 and 40 mg/kg) had a noteworthy effect on hemodynamic parameters, reduction of oxidative capacity, and reduction of heart rate (47-48). Jahanbakhsh *et al*. in 2012, reported that the protective role of crocin had been observed on severe arrhythmia caused by cardiac perfusion in rats, and crocin was shown to recover the level of anti-oxidant enzymes in the heart tissue of rats with ischemic reperfusion (49). In a study by Goyal *et al*. in 2010 on rats, the protective role of crocin in controlling the formation of lipid peroxide was shown to improve the anti-oxidants system in the Isopterenol-induced toxicity (50). The study of Hossein Zadeh *et al*. described that the water-alcohol extract of saffron could significantly reduce pulse and cardiac contractility in guinea pigs (51). In a study by Khouri *et al*. in 2006, the hydroalcoholic extract of saffron had a protective role on the ventricular atrial node in treating supraventricular arrhythmias. Different doses of this extract increased the baseline electrophysiological parameters (ventricular conduction time, functional stress irritation time) (52).

## Conclusion

Cardiac ischemia causes abnormal hemodynamic factors of the heart, which are exacerbated by PM10 and further reduce the heart’s contractile strength. Increased oxidative stress due to PM10 is probably one of the important reasons for these changes. This study suggests that the use of anti-oxidants such as crocin improves the cardiovascular adverse effects of myocardial ischemia and PM10 exposure.

## Authors’ Contributions

DM and RE Conceived the study and design; DM, RE, RM, and MSA Performed data processing, collection, and experiments; DM and RE Analyzed data and prepared the draft manuscript; RM Critically revised the paper; DM Supervised the research; DM, RE, RM, BM, GGh, and MSA Approved the final version to be published.

## Conflicts of Interest

The authors declare no conflicts of interest.
